# Effect of Internal Structural Design on Stress Distribution in 3D-Printed Subperiosteal Implants Under Mechanical Loading

**DOI:** 10.3390/bioengineering13030368

**Published:** 2026-03-20

**Authors:** Ádám Vörös, Balázs Lőrincz, János Kónya, Ibolya Zsoldos

**Affiliations:** 1 Department of Materials Science and Technology, Széchenyi István University, 9026 Győr, Hungary; 2 Dent Art Technik Kft., 9024 Győr, Hungary; janos@dentarttechnik.hu

**Keywords:** subperiosteal implant, implant deformation, finite element analysis, additive manufacturing, computed tomography, mandibular implant, lattice structures

## Abstract

Custom-made subperiosteal implants are increasingly used in clinical cases where significant bone loss due to trauma or disease renders conventional endosseous implant placement unfeasible. This study investigated how different internal structural designs affect the deformation and stress distribution in mandibular subperiosteal implants under clinically relevant loading conditions. An idealized implant geometry was defined based on average human mandibular dimensions, and four configurations with identical outer shape and connection features were created, differing only in sidewall architecture (solid, top-relieved, top-relieved with lateral perforations, and top-relieved lattice framework). All specimens were manufactured by metal additive manufacturing and evaluated using cone-beam computed tomography (CBCT). Mechanical testing was performed in two stages: (i) cyclic loading consisting of 500 bite cycles at an overall force of ~326–350 N and (ii) a single static high-load event of 2000 N, applied parallel to the fixation pin axes. CT datasets acquired before and after each stage were compared to detect permanent deformation. No measurable residual deformation was identified in any configuration; the only observed macroscopic change was an adhesive-bond limitation in one case, rather than structural yielding of the implant. Finite element analysis further supported these findings by identifying localized stress concentrations mainly at the implant–prosthetic interface and by revealing the load-transfer zones that govern the mechanical response. Overall, the results indicate that lightweight, perforated, and lattice-based internal designs can preserve global structural integrity across physiological and supra-physiological load ranges while enabling design optimization to improve stress distribution.

## 1. Introduction

Thanks to interdisciplinary collaboration among healthcare, medical sciences, and engineering fields such as materials science and mechanical engineering, it has become possible to restore patients’ original masticatory and biting functions following trauma or disease. The replacement of missing teeth and the restoration of masticatory function have been pursued by humankind since ancient times; however, the available materials and medical knowledge did not permit durable, high-quality dental restorations. Significant progress became possible only after the nineteenth century, driven by the introduction of advanced biomaterials such as titanium, cobalt–chromium alloys, stainless steels, and later ceramic and polymer-based materials. In parallel, major developments in pain management—including modern anesthesia techniques—as well as the widespread use of antibiotics, substantially reduced surgical risks, particularly the incidence of postoperative infections and sepsis. As a result, dental and maxillofacial implantology experienced rapid and continuous advancement [1,2,3,4].

In modern dental implantology, implant systems can be classified into three main categories based on their geometry, dimensions, and—most importantly—their method of fixation to the mandible or maxilla. The first group comprises endosteal implants, which are screw-shaped devices inserted directly into the jawbone; these are the most commonly used for replacing single teeth or small edentulous segments. The second category includes transosteal implants, which traverse the entire thickness of the jawbone and are typically applied in specific clinical cases requiring enhanced mechanical anchorage. The third category consists of subperiosteal implants, which, unlike endosteal systems, are not placed within the bone but are positioned on the surface of the bone beneath the periosteum [5,6].

**Figure 1:**
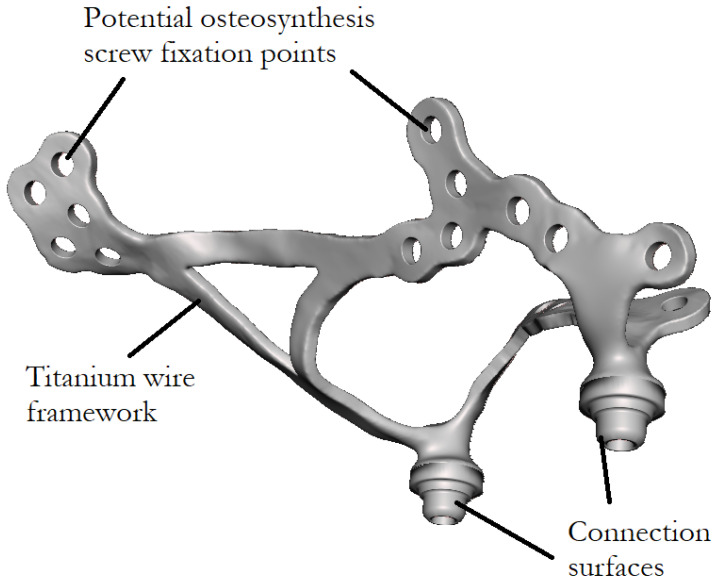
Custom-made maxillary subperiosteal implant.

**Figure 2:**
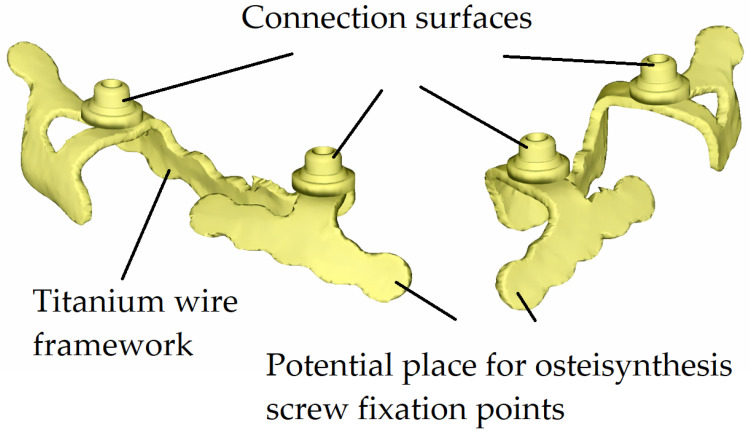
Custom-made mandibular subperiosteal implant.

An example of a custom-made subperiosteal implant investigated in this study is shown in Figure 1, representing a maxillary implant designed for fixation to the upper jaw. In contrast, Figure 2 presents a mandibular implant intended for fixation to the lower jaw.

These custom-made implants were previously manufactured based on physical impressions; however, nowadays they are produced using cone-beam computed tomography (CBCT) data. The main steps of the implant manufacturing workflow are as follows:

- Acquisition of a physical impression or CBCT scan.
- Development of a treatment and design concept through collaboration between the implant designer and the dental clinician.
- Computer-aided design (CAD) of the implant geometry.
- Review and approval of the implant design by the dentist or oral surgeon.
- Generation of a printable three-dimensional model.
- Additive manufacturing of the implant using metal 3D printing technology.
- Machining of functional connection surfaces and threads using five-axis milling.
- Heat treatment of the additively manufactured component.
- Surface finishing of the printed implant, including sandblasting.
- Removal of support structures from the printed implant, followed by final manual finishing using hand tools.
- Trial pre-assembly of the completed implant system [7,8,9,10,11,12].

For endosteal implants, the qualification and testing requirements are defined in ISO 14801:2016 [13]. In contrast, subperiosteal implants lack a standardized qualification protocol owing to their patient-specific, custom-made nature. The present study aims to investigate the effect of different additively manufactured infill structures on the internal stress distribution and permanent deformation of subperiosteal implants under various loading conditions. Based on the results obtained, design-oriented recommendations are proposed to support implant designers in selecting appropriate infill structures for different regions of the implant [14].

Lattice-based frameworks, perforated configurations, and top-relieved structural designs (see Table 1) may provide several potential advantages and beneficial effects, including:

- Improved internal stress distribution under mechanical loading.
- Enhanced manufacturability by metal additive manufacturing, including reduced warping caused by thermal gradients.
- Reduced material consumption.
- Shorter manufacturing time.
- Potentially improved osseointegration characteristics.
- Reduced volume of implanted foreign material, which may contribute to a more favorable long-term bone response.

**Table 1:** Main parameters of the four tested specimens.

No.	Design	Weight [g]	Surface Area [mm^2^]	Volume [mm^3^]	Infill Density ^1^ [%]
1	Lattice framework	1.18	770.37	265.38	30.66
2	Perforated	2.56	1659.73	577.91	66.77
3	Top-relieved	3.16	1763.43	713.33	82.41
4	Solid	3.83	2109.39	865.59	100

^1^ If the solid configuration is considered as the 100% reference.

Among these factors, the latter two are considered the most important. Compared to solid designs, lattice-based or perforated implant structures facilitate more effective osseointegration. Osseointegration is the biological process by which living bone tissue gradually grows onto and into the surface features and internal pores of an implanted material, forming a direct structural and functional connection between the bone and the implant. As demonstrated by Wei Wang et al., porous and lattice-structured titanium implants significantly enhance bone ingrowth by providing increased surface area and interconnected pathways for tissue penetration [15,16,17].

In addition to improved osseointegration, lattice-based implant designs may also mitigate stress shielding. Stress shielding occurs when an implant with excessively high stiffness carries a disproportionate share of the applied load, reducing mechanical stimulation of the surrounding bone and leading to bone resorption over time. According to the findings of Minku et al., optimized porous lattice structures can tailor the effective elastic modulus of titanium implants closer to that of natural bone, thereby improving load transfer and promoting long-term implant stability [18].

For the experimental investigation, a custom-designed fixture was developed to hold the test specimens securely. The connection between the base support components and the tested implants was ensured by adhesive bonding. The implants were adhered to the mandibular model using a two-component Pattex Repair Universal epoxy adhesive (Henkel AG & Co. KGaA, Düsseldorf, Germany). After curing, the adhesive exhibited a tensile strength of approximately 130 kg/cm^2^ (≈13 MPa) at 24 °C, which was selected to approximate the mechanical constraint provided by osseointegration between the implant and the surrounding bone [19].

The base support components and the mandibular model were manufactured by stereolithography (SLA) using a Form 3B + 3D printer (Formlabs Inc., Somerville, MA, USA) from Tough 2000 Resin V1 (Formlabs Inc., Somerville, MA, USA). The mandibular specimens were produced by PAB Kft. (Budapest, Hungary) based on their in-house measurements and the material property data provided in the manufacturer’s material datasheet. After post-processing, the material exhibited a Young’s modulus of approximately 1.2 GPa and a Poisson’s ratio of 0.35, representing the compliant mechanical behavior of mandibular bone. The tested implant specimens were fabricated from Ti–6Al–4V, with mechanical properties reflecting the applied heat treatment and surface finishing, including a Young’s modulus of 113 GPa, a Poisson’s ratio of 0.342, a yield strength of approximately 790 MPa, and an ultimate tensile strength of approximately 860 MPa [20,21].

Through this work, we seek to take a step toward establishing standardized, mechanically sound, and clinically reliable evaluation approaches for patient-specific subperiosteal implants.

## 2. Materials and Methods

The mandible and the reinforcing subperiosteal implant are predominantly subjected to compressive loading between the upper and lower dentition. It was hypothesized that the different structural regions of the implant do not contribute equally to load bearing and therefore require function-specific design considerations. Accordingly, optimized lightweight internal structures were expected to maintain global structural integrity while providing stress-distribution characteristics comparable to those of the fully solid configuration over the investigated load range. The objective was to map the local mechanical stress distribution throughout the entire implant volume, identify minimally and maximally loaded regions, and define structurally favorable surface and lattice configurations in the titanium framework accordingly.

Due to the patient-specific nature of subperiosteal implants and the variability in individual clinical requirements, a fully objective comparison between different implant designs is inherently challenging. To simplify the investigation and ensure comparability, the present study focused exclusively on mandibular subperiosteal implants. This choice was motivated by the availability of an existing experimental test rig specifically developed to evaluate mandibular implant systems. The test setup and its operational principle applied in the present study are identical to those described in our previously published experiments [22].

A general and simplified reference model was created specifically for the present investigations, in which the curved regions, the straight structural segments, and the areas surrounding the connection posts are clearly distinguishable. This configuration was intentionally designed to enable objective comparison of differently structured, lightweight internal configurations. The overall shape and geometric dimensions of the implant—including width, length, height, filet radii, connection cones, and the spacing of vertical posts—were defined based on average mandibular anatomical dimensions. The design parameters were established in consultation with dental implant designers from Dent-Art Technik, a Hungarian dental manufacturing company headquartered in Győr, to ensure clinical relevance and realistic geometry.

**Figure 3:**
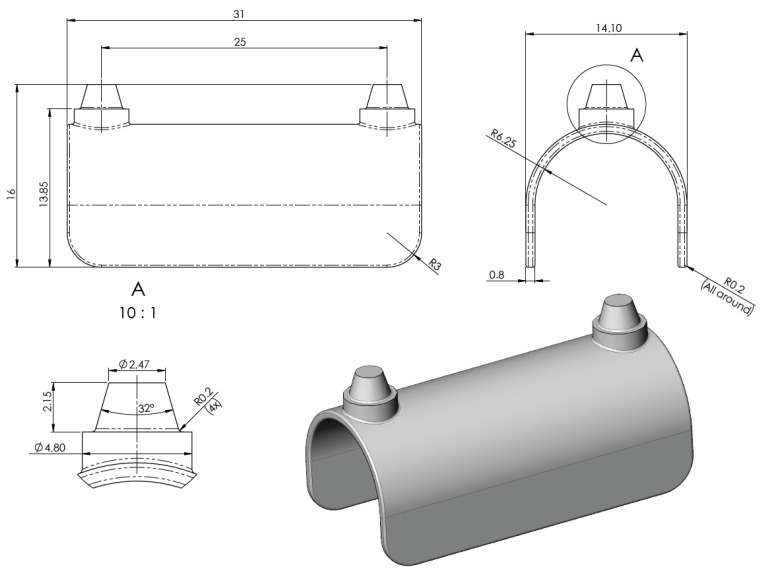
Simplified mandibular subperiosteal implant model.

The initial reference model of the simplified implant geometry is shown in Figure 3.

The model shown in the design drawings was used as the baseline reference geometry and was subsequently considered the 100% infill configuration. Based on this reference model, three additional implant variants with different lightweight (relieved) internal structures were developed. The main parameters of the four tested implant configurations are summarized in Table 1.

**Figure 4:**
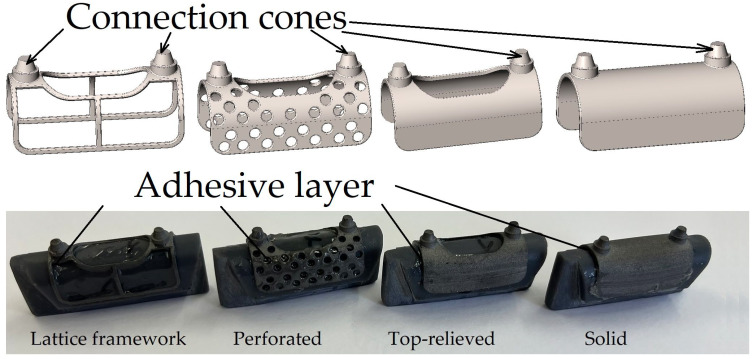
Test specimens before testing.

The four test specimens prepared for the experimental investigation are shown in Figure 4. The specimens were manufactured at Dent-Art-Technik Kft. by laser sintering using a TruPrint 1000 Basic Edition metal additive manufacturing system (TRUMPF SE + Co. KG, Ditzingen, Germany). The material used was POWDERRANGE^®^ Ti64 supplied by Carpenter Additive (Carpenter Technology Corporation, Philadelphia, PA, USA).

After adhesive bonding, each test specimen was mounted into a mandibular model manufactured from Tough 2000 Resin V1. Subsequently, cone-beam computed tomography (CBCT) scans were acquired for each assembled specimen to obtain reference datasets for later comparison. In addition, micro-CT measurements were performed using a Yxlon Modular CT system (YXLON International GmbH, Hamburg, Germany). A tube voltage of 225 kV and a flat-panel detector were used. The combination of the 2048 × 2048-pixel matrix detector, the dimensions of the implant models, and the technical parameters of the CT system yielded a spatial resolution of 19 μm. The acquired projection data were reconstructed using Volume Graphics StudioMax 2025.3 software (Volume Graphics GmbH, Heidelberg, Germany).

Following the baseline CBCT acquisition, the specimens underwent the first loading cycle, after which a second CBCT scan was performed. Subsequently, a second loading cycle was applied, followed by an additional CBCT scan. The acquired CBCT datasets were later compared with the reference scans to identify potential permanent deformations induced by mechanical loading.

The first loading cycle was designed to simulate 500 average mastication cycles under physiological biting conditions. During this loading protocol, the target load was set to 160 N per connection cone; however, force measurements performed on the test rig indicated an average applied load of approximately 163 N per cone, resulting in a total applied load of 326 N. In contrast, the second loading cycle consisted of a single high-load event, corresponding to approximately three to four times the average maximum human bite force. In all test cases, the applied loads were distributed over the upper surfaces of the two main connection cones and aligned parallel to the cones’ longitudinal axes.

**Figure 5:**
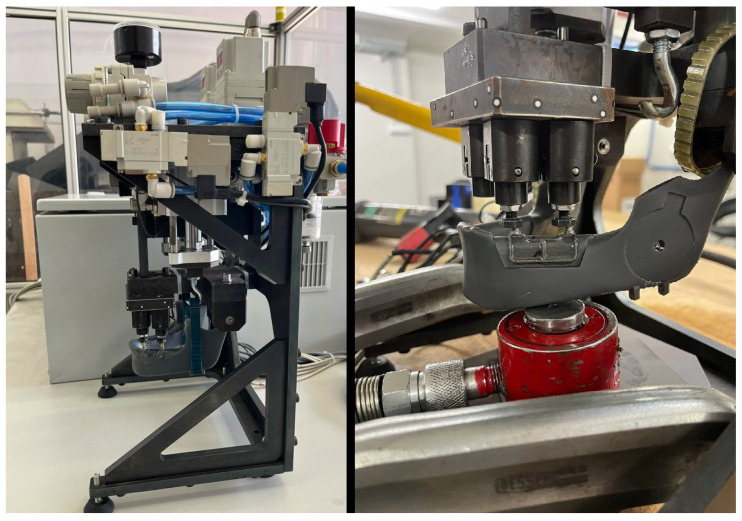
Two different testing configurations.

During the first loading cycle, the pneumatic biting test apparatus shown on the left side of Figure 5 was employed. For the second loading cycle, the same test setup was used; however, because the pneumatic system could not generate the required load of approximately 2000 N, the two pneumatic actuators were replaced with a hydraulic compression cylinder. In this configuration, the hydraulic cylinder (shown in red) is positioned beneath the mandibular model, as depicted on the right side of Figure 5 [23].

The main parameters of the two loading cycles are summarized in Table 2. Between two consecutive loading (biting) cycles, only a single opening phase was applied. The duration of this phase depended on the response speed of the pneumatic system and the control unit and was less than 0.5 s. No additional relaxation time was introduced between the loading cycles.

To eliminate the limitations associated with physical load cases, enable rapid and cost-effective testing, and visualize material behavior, numerical simulations (i.e., virtual testing) were performed in addition to the experimental investigations. The applied simulation approach was the finite element method (FEM), a numerical technique widely used to analyze and predict the mechanical behavior of complex structures. Since biomechanical processes are governed by the same physical laws as classical mechanical systems, FEM-based simulations are well-suited for this type of analysis [24].

**Figure 6:**
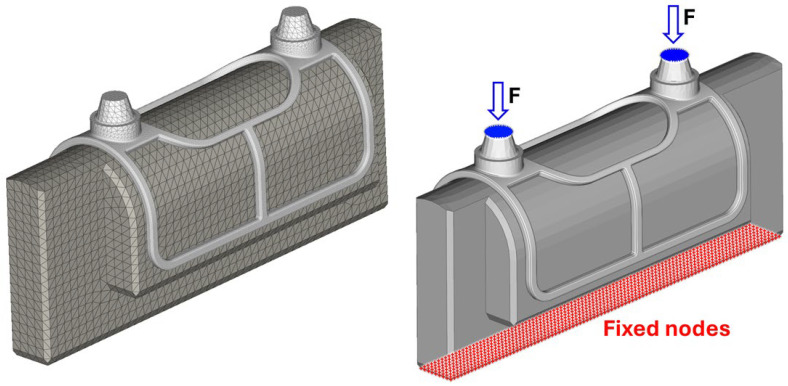
Finite element mesh and model build-up.

During modeling, the test environment was virtually reconstructed using Abaqus 2026 FD01 software (Dassault Systèmes SE, Vélizy-Villacoublay, France), as shown on the left side of Figure 6. The numerical model included a simplified segment of the mandible and the implant mesh with bushings. The epoxy adhesive layer between the components was modeled as a cohesive contact formulation in a simplified manner, accounting for the adhesive’s stiffness. However, damage and failure of the adhesive material were not modeled; therefore, the numerical simulations do not capture adhesive failure scenarios.

The finite element mesh consisted of 158,660 second-order tetrahedral (C3D10) elements. The applied load was introduced as a uniformly distributed concentrated force acting on the bushing nodes in the vertical direction, while the lower nodes of the mandibular segment were fully constrained in the X, Y, and Z directions (right side of Figure 6).

The titanium material was modeled using a bilinear elasto-plastic material law, while the mandible was modeled using Hooke’s law, assuming linear elastic behavior. The corresponding material parameters used in the numerical simulations are summarized in Table 3. Although the Tough 2000 mandible material is significantly weaker than titanium, it was subjected exclusively to compressive stresses; therefore, damage initiation is expected to occur only at higher load levels. Consequently, the elastic modeling of the mandible represents a reasonable simplification within the investigated load range and is consistent with the assumed failure limits [20].

## 3. Results

### 3.1. CT-Based Deformation Results

**Figure 7:**
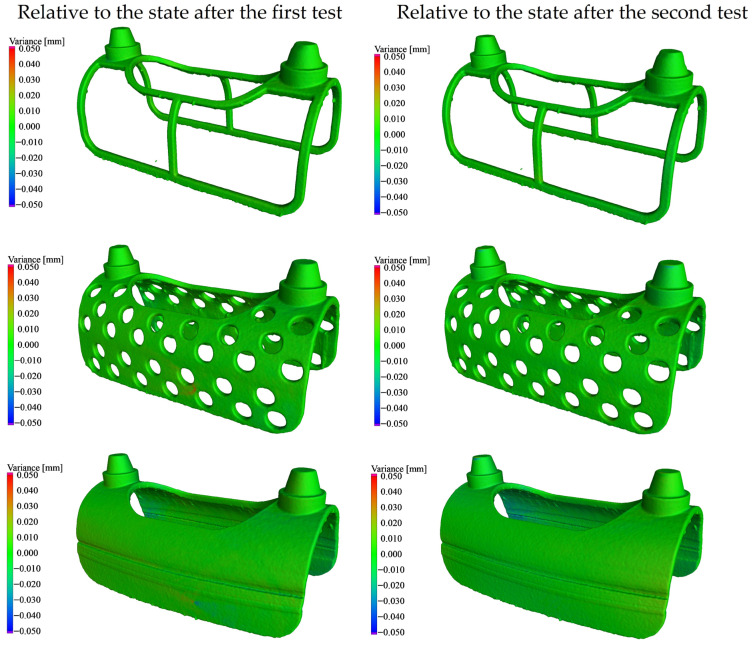
Comparison of CT reconstruction models before and after loading in structured architectures.

CT scans acquired before and after the mechanical tests were compared. Based on this evaluation, it can be concluded that for Samples 1–3, no permanent deformation was observed exceeding the expected measurement accuracy of the CT system (±0.02 mm). The comparative analysis of these three cases is presented in Figure 7.

**Figure 8:**
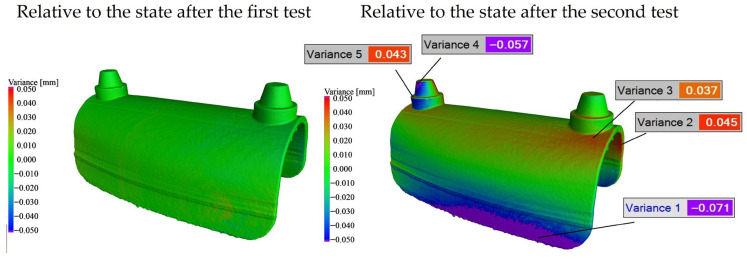
Comparison of CT reconstruction models before and after loading the solid structure.

The only case in which measurable permanent deformation was detected was the fully solid specimen (Sample 4). In this case, no permanent deformation was observed after the first test cycle; however, following the second test cycle, residual deformation became detectable. The comparative CT-based evaluation of Sample 4 is shown in Figure 8.

This behavior can be attributed to the failure of the adhesive joint that ensured the connection between the base holder and the fully solid specimen during the tests. As a result of the loss of proper support, the specimen was able to deform. In the case of fully solid printed specimens, warping during the manufacturing process limited geometric accuracy; consequently, the gap between the base holder and the specimen was entirely filled by the adhesive layer. Once this adhesive connection partially or completely failed (fully on one side and partially on the other), the specimen could undergo displacement and permanent deformation.

### 3.2. Finite Element Result

During the simulation, the component was subjected to a continuously increasing distributed load applied symmetrically on each side. The load magnitude increased from 0 to 2000 N per side over the course of the simulation. The analysis was carried out using a time-independent static nonlinear step (NLGEOM = YES) with the Abaqus Implicit solver. Among the output variables, particular attention was paid to the first principal stress and the accumulated equivalent plastic strain (PEEQ) in the titanium material, evaluated at the finite element integration points. According to the simulation results, the average load applied during the measurement did not induce any permanent deformation in the implant mesh.

**Figure 9:**
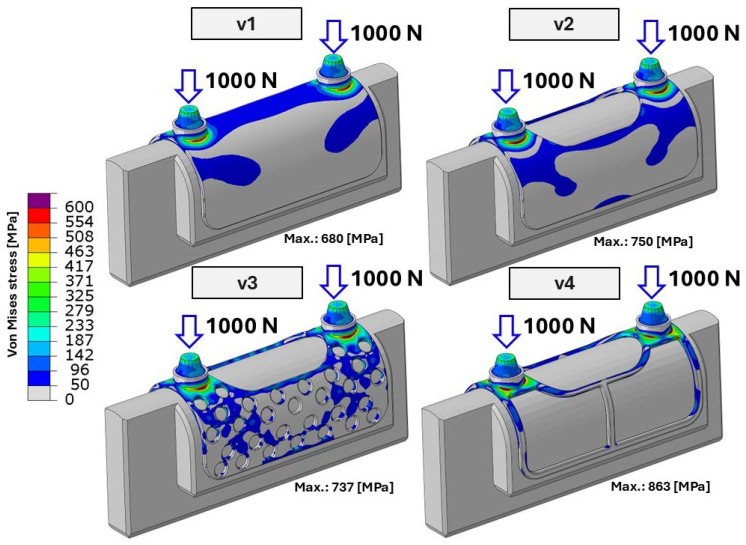
Maximum von Mises stress distribution of the tested specimens at 1000 N. V1–V4 denote the individual test specimens, while the arrows indicate the direction of the applied load.

During the first loading cycle (500 bite cycles with a load amplitude of 326 N), none of the investigated specimens reached local stress levels sufficient to cause macroscopic permanent (plastic) deformation. Although in certain highly localized regions the stress values slightly exceeded the material yield limit, this effect remained confined to a very small volume and did not result in detectable permanent deformation at the structural level. Consequently, no residual deformation could be identified on the post-test CT scans. The evaluated local stress distribution and the corresponding maximum local stress values for the four different specimens during the second test cycle are presented in Figure 9.

**Figure 10:**
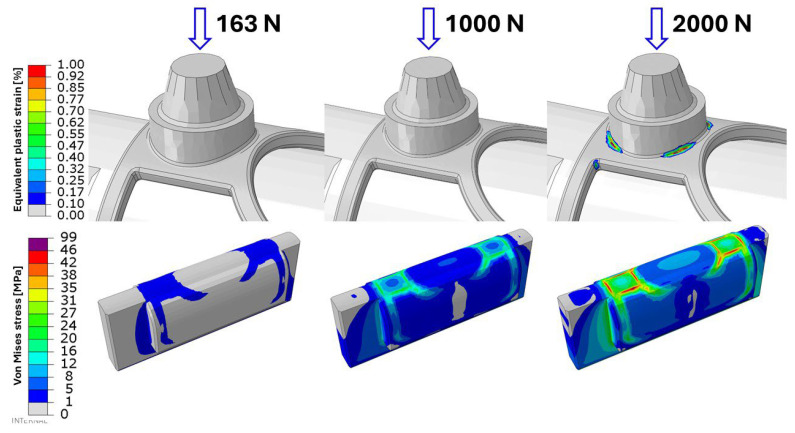
Stress and strain response of the most critical specimen (Sample 4) under increasing load levels. The arrows indicate the direction of the applied load.

As shown in Figure 10, the evaluated stress and strain distributions for the most critical specimen (Sample 4) are presented for both the first and second test cycles. In addition, the figure illustrates the predicted structural response of the implant mesh under a substantially increased loading scenario, corresponding to a total applied load of 4000 N (2000 N per side).

It should be emphasized that permanent deformation was observed only under this extreme loading condition, which was not applied during the experimental tests and is likely unrealistic in practical scenarios. At this load level, as also indicated in the lower part of Figure 10, the yield limit of the polymer clamping component is exceeded. Consequently, neither the experimental setup nor the corresponding finite element model is suitable for reliable measurements at such high load levels.

Based on these observations, a mechanically stiffer clamping system would be required to investigate the loading conditions of this magnitude. However, such extreme forces are not representative of physiological masticatory loads in dental implantology and therefore fall outside the practical scope of the present study.

## 4. Discussion

**Figure 11:**
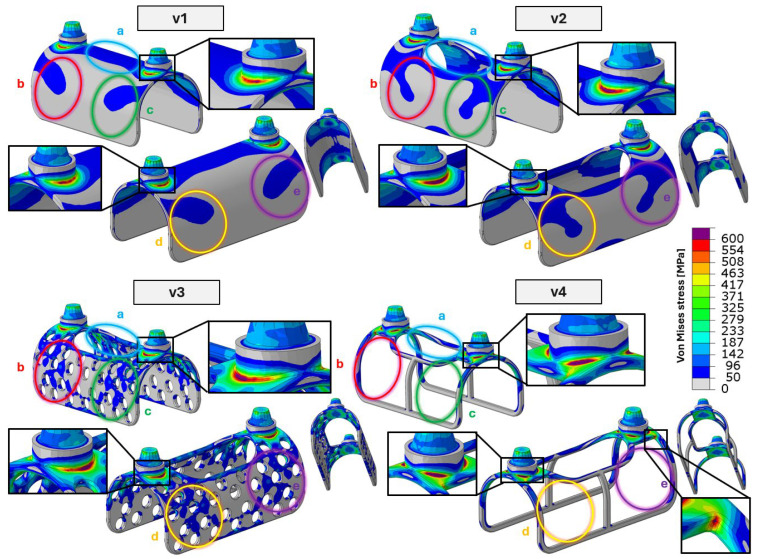
Critical cross-sections and load-transfer zones. V1–V4 denote the individual test specimens, while a–e indicate the load-bearing regions of the implant; the highlighted detail views show the critical cross-sections.

Based on the finite element analysis (FEA) results, the exact surfaces responsible for load transfer between the implant and the base support were identified, where the base support represents the mandible in the clinical scenario. As shown in Figure 11, the locations and extents of the critical cross-sections, as well as all main load-transfer zones, are clearly visible. For all investigated specimens, the most critical regions were found at the interfaces between the conical fixation pins—used to attach the prosthetic superstructure—and the implant’s lattice structure.

In addition to the implant–prosthetic interface, five primary load-transfer zones, denoted as regions (a)–(e), can be identified. The critical cross-sections associated with these load-transfer zones are visible and highlighted for all four investigated implant configurations (and not only for the solid reference specimen) in Figure 11. Among the identified regions, the upper region (a) exhibits the largest contact area in the solid configuration. In practical implant design, this region is commonly lightweighted to reduce stress shielding caused by insufficient mechanical stimulation, which may otherwise lead to bone resorption. Consequently, in lightweight configurations, the relative mechanical relevance of the remaining regions (b)–(e) increases.

In the third specimen, the introduction of perforations does not result in additional mechanical disadvantages. On the contrary, the improved stress distribution reduces the maximum local stress from 750 MPa to 737 MPa.

In the fourth test specimen, local stresses exceeding the material yield strength (~790 MPa) are observed, with peak stress values reaching 863 MPa, which also exceed the material’s guaranteed ultimate tensile strength. These extreme stresses, however, are confined to very small regions located at the critical cross-sections of the lattice structure, as indicated in Figure 11. The spatial extent of the plastically affected zones is therefore highly limited. Since the applied loading consisted of a single high-load event rather than cyclic loading, the global structural response remained predominantly elastic. Consequently, no permanent deformation could be detected based on the comparative CT analyses.

This behavior is consistent with established theoretical interpretations of localized plasticity in metallic structures. Rice demonstrated [28] that stress concentrations at geometric discontinuities may induce highly confined plastic zones without leading to global structural deformation. Furthermore, as discussed by Suresh [25], localized yielding under non-cyclic loading conditions does not necessarily result in measurable permanent deformation when the plastically affected volume remains limited and does not propagate. Accordingly, the absence of detectable permanent deformation in the CT-based evaluation is consistent with the localized nature of the stress peaks predicted by the numerical simulations.

Based on the CT evaluation, no permanent deformation was detected in the tested specimens, except for a single case in which the specimen’s adhesive bond reached its maximum load-bearing capacity. When these observations are considered together with the FEA results, no significant differences in the mechanical behavior of implants with different lattice structures were observed, provided that the applied load was aligned parallel to the fixation pins and did not exceed a total load of 2000 N (corresponding to 1000 N per side). The selected load levels are consistent with reported physiological and biomechanical limits of the human mandible. Previous studies indicate that normal masticatory bite forces typically range around 300–400 N under physiological conditions. Experimental and numerical investigations further suggest that localized microdamage or microcrack formation in mandibular bone may occur at elevated load levels on the order of approximately 1500 N, particularly in weakened or compromised bone, while macroscopic mandibular fracture generally requires substantially higher forces, often exceeding 2500–3000 N under controlled loading conditions. Accordingly, the limitations of the present study are primarily related to the maximum applicable load, which was constrained by the mechanical properties of the mandible model and the base support, both manufactured from polymer material. However, applying substantially higher loads would also increase the risk of damage to the real mandibular bone in clinical scenarios and would therefore not be physiologically meaningful. In addition, the investigation focused on the magnitude of the applied biting forces rather than long-term cyclic loading. The specimens were subjected to a total of 500 mastication cycles under physiological load conditions, followed by a single high-load event of 2000 N. High-cycle fatigue behavior under extended loading conditions was beyond the scope of the present study and will be addressed in future work [23,29,30].

Based on experimental observations, numerical results, and the supporting biomechanical literature, the applied loading protocol can therefore be considered appropriate and representative for evaluating the mechanical response of the investigated implant configurations within clinically relevant limits. The experimental and numerical results obtained confirm the proposed hypothesis: the different structural regions of the implant contribute unequally to load bearing, and optimized lightweight internal configurations can preserve global mechanical integrity within the investigated load range.

### Limitations and Future Perspectives

The present study has several limitations that should be acknowledged. First, the applied loading protocol consisted of 500 physiological bite cycles followed by a single high-load event of 2000 N. Although this approach allowed the evaluation of structural integrity within clinically relevant limits, it does not represent long-term fatigue behavior under lifelong functional loading conditions. High-cycle fatigue testing over several million loading cycles was not possible with the current experimental setup.

Second, the applied loads were aligned parallel to the longitudinal axes of the connection cones and fixation pins. While this configuration represents a simplified, clinically relevant axial loading scenario, it does not account for non-axial or inclined load components that may occur in vivo during complex mastication movement.

Third, the mechanical properties of the polymer-based mandibular model and the clamping system limited the maximum load level that could be applied. At substantially higher loads, deformation of the support structure may occur, restricting the reliability of measurements under extreme loading conditions.

To overcome these limitations, a new computer-controlled fatigue test bench is currently being designed. The conceptual and engineering plans have already been completed, and construction of the system is planned soon. The new setup will enable high-cycle fatigue testing (2–5 million loading cycles at controlled frequencies of approximately 5–15 Hz) on various implant configurations.

Future investigations will include extended cyclic loading on multiple implant types, followed by microscopic and CT-based analyses to detect fatigue-related microdamage, crack initiation, or structural degradation. Furthermore, non-axial loading conditions will be examined in accordance with ISO 14801:2016, in which the applied force is applied at a defined 30° inclination relative to the implant axis and combined with dynamic fatigue loading. This approach will provide a more comprehensive mechanical evaluation under clinically relevant worst-case loading scenarios [13].

## 5. Conclusions

- CT-based deformation measurements and finite element simulations consistently demonstrated that none of the implant configurations investigated exhibited permanent deformation under clinically relevant loading conditions up to 2000 N, regardless of the applied internal infill strategy.
- Load-transfer analysis revealed that critical stresses concentrate primarily at the implant–prosthetic interface, while structural regions located farther from this zone experience significantly lower stress levels and therefore do not govern global static integrity.
- In high-load-transfer regions, sufficient material presence is essential; however, perforated designs can safely replace fully solid structures, while in low-stress regions, extensive lightweighting may be applied without compromising mechanical performance.
- Lightweight, perforated, and lattice-based configurations maintain global mechanical stability while potentially improving stress distribution, reducing stress shielding, and enhancing osseointegration. Consequently, the titanium reinforcement mesh can be optimized primarily for biological rather than load-bearing considerations.

## Figures and Tables

**Table 2:** Testing configuration parameters.

No.	Number of Bites [pcs]	Right and Left Bite Force [N]	Full Bite Force [N]	Bite Length [s]
1	500	163	326	2
2	1	1000	2000	10

**Table 3:** Applied material properties [3,11,25,26,27].

Property	Mandible (Tough 2000)	Implant Mesh (Ti6Al 4V)
Young modulus	1.2 GPa	113 GPa
Poisson ratio	0.35	0.342
Yield strength	not modeled	790 MPa
Ultimate strength	not modeled	860 MPa
Elongation at break	not modeled	15%

## Data Availability

The original contributions presented in the study are included in the article; further inquiries can be directed to the corresponding authors.

## References

[1] Abraham C.M. (2014). A Brief Historical Perspective on Dental Implants, Their Surface Coatings and Treatments. Open Dent. J..

[2] Marin E., Lanzutti A. (2025). History of Metallic Orthopedic Materials. Metals.

[3] Geetha M., Singh A.K., Asokamani R., Gogia A.K. (2009). Ti Based Biomaterials, the Ultimate Choice for Orthopaedic Implants—A Review. Prog. Mater. Sci..

[4] Froes F.H. (2018). Titanium for Medical and Dental Applications—An Introduction. Titanium in Medical and Dental Applications.

[5] Łoginoff J., Majos A., Elgalal M. (2024). The Evolution of Custom Subperiosteal Implants for Treatment of Partial or Complete Edentulism in Patients with Severe Alveolar Ridge Atrophy. J. Clin. Med..

[6] Misch C.E. (2015). Dental Implant Prosthetics.

[7] Meglioli M., Naveau A., Macaluso G.M., Catros S. (2020). 3D Printed Bone Models in Oral and Cranio-Maxillofacial Surgery: A Systematic Review. 3D Print. Med..

[8] Al-Nawas B., Bär A.-K. (2025). Virtual Surgical Planning and Customized Subperiosteal Implants: A Systematic Review. Int. J. Oral Maxillofac. Surg..

[9] Joda T., Ferrari M., Gallucci G.O., Wittneben J., Brägger U. (2017). Digital Technology in Fixed Implant Prosthodontics. Periodontol. 2000..

[10] Vercruyssen M., Laleman I., Jacobs R., Quirynen M. (2015). Computer-supported Implant Planning and Guided Surgery: A Narrative Review. Clin. Oral Implants Res..

[11] Murr L.E., Gaytan S.M., Ramirez D.A., Martinez E., Hernandez J., Amato K.N., Shindo P.W., Medina F.R., Wicker R.B. (2012). Metal Fabrication by Additive Manufacturing Using Laser and Electron Beam Melting Technologies. J. Mater. Sci. Technol..

[12] Nedelcu L., Sirbu I., Sirbu V.D., Custura A.M., Radu A., Nastasie V. (2024). Custom-made 3D Printed Subperiosteal Implant for Restoration of Severe Atrophic Jaw: A Case Report. Clin. Case Rep..

[13] Dentistry—Implants—Dynamic Fatigue Test for Endosseous Dental Implants. https://www.iso.org/standard/61997.html.

[14] Vanaclocha V., Atienza C., Vanaclocha A., Peñuelas A., Gómez-Herrero J., Pérez-Carrió F., Diego-Leyda J.A., Sáiz-Sapena N., Vanaclocha L. (2025). New Subperiosteal Dental Implant Design with Finite Element Analysis and Mechanical Validation: A Design Validation Study. Materials.

[15] Wang W., Xiong Y., Zhao R., Li X., Jia W. (2022). A Novel Hierarchical Biofunctionalized 3D-Printed Porous Ti6Al4V Scaffold with Enhanced Osteoporotic Osseointegration through Osteoimmunomodulation. J. Nanobiotechnol..

[16] Smeets R., Stadlinger B., Schwarz F., Beck-Broichsitter B., Jung O., Precht C., Kloss F., Gröbe A., Heiland M., Ebker T. (2016). Impact of Dental Implant Surface Modifications on Osseointegration. Biomed Res. Int..

[17] Sattary M., Rafienia M., Kazemi M., Salehi H., Mahmoudzadeh M. (2019). Promoting Effect of Nano Hydroxyapatite and Vitamin D3 on the Osteogenic Differentiation of Human Adipose-Derived Stem Cells in Polycaprolactone/Gelatin Scaffold for Bone Tissue Engineering. Mater. Sci. Eng. C.

[18] Minku, Jain T., Ghosh R. (2024). Comparative Analysis of Tissue Ingrowth in Printable Porous Lattice Structured Implants: An in Silico Study. Materialia.

[19] Henkel Magyarország Kft (2025). Pattex Repair Universal—Two-Component Epoxy Adhesive: Technical Data Sheet.

[20] Kamali A., Sarabian M., Laksari K. (2023). Elasticity Imaging Using Physics-Informed Neural Networks: Spatial Discovery of Elastic Modulus and Poisson’s Ratio. Acta Biomater..

[21] Tough 2000 Resin for Rugged Prototyping. https://formlabs-media.formlabs.com/datasheets/2001340-TDS-ENUS-0P.pdf.

[22] Talabérné K.K., Vörös Á., Zsoldos I. (2024). Mechanical Fatigue Test of Individual Dental Implants. Proceedings of the Advances in Transdisciplinary Engineering.

[23] Levartovsky S., Peleg G., Matalon S., Tsesis I., Rosen E. (2022). Maximal Bite Force Measured via Digital Bite Force Transducer in Subjects with or without Dental Implants—A Pilot Study. Appl. Sci..

[24] Gustafson H.M., Cripton P.A., Ferguson S.J., Helgason B. (2017). Comparison of Specimen-Specific Vertebral Body Finite Element Models with Experimental Digital Image Correlation Measurements. J. Mech. Behav. Biomed. Mater..

[25] Suresh S. (1998). Fatigue of Materials.

[26] Form Cure V1 Time and Temperature Settings. https://s3.amazonaws.com/servicecloudassets.formlabs.com/media/Finishing/Post-Curing/115001414464-Form%20Cure%20Time%20and%20Temperature%20Settings/FormCurePost-CureSettings.pdf.

[27] PowderRange Ti64 Applicable Specifications: ASTM F3001. https://www.carpenteradditive.com/hubfs/Carpenter%20Additive/Resources/Datasheets/PowderRange_Ti64_Datasheet.pdf.

[28] Rice J.R. (1968). A Path Independent Integral and the Approximate Analysis of Strain Concentration by Notches and Cracks. J. Appl. Mech..

[29] Cao X., Xiao S., Shen C., Fan Y. (2025). Microdamage in Biological Hard Tissues and Its Repair Mechanisms. Biomed. Eng. Online.

[30] Unnewehr M., Homann C., Schmidt P.F., Sotony P., Fischer G., Brinkmann B., Bajanowski T., DuChesne A. (2003). Fracture Properties of the Human Mandible. Int. J. Legal Med..

